# Feasibility and acceptability of 7-day smartphone-based, activity-triggered Ecological Momentary Assessment among low-income older adults

**DOI:** 10.1093/geroni/igaf151

**Published:** 2026-01-07

**Authors:** Olivia S Malkowski, Genevieve F Dunton, Nick P Townsend, Mark J Kelson, Max J Western

**Affiliations:** Centre for Motivation and Behaviour Change, Department for Health, University of Bath, Bath, United Kingdom; Department of Population and Public Health Sciences, Keck School of Medicine, University of Southern California, Los Angeles, California, United States; Centre for Exercise, Nutrition and Health Sciences, School for Policy Studies, University of Bristol, Bristol, United Kingdom; Institute of Data Science and Artificial Intelligence, Department of Mathematics, University of Exeter, Exeter, United Kingdom; Centre for Motivation and Behaviour Change, Department for Health, University of Bath, Bath, United Kingdom

**Keywords:** Socioeconomic status, Recruitment, Ambulatory assessment, Accelerometer, Exercise/Physical activity

## Abstract

**Background and Objectives:**

Smartphone-based Ecological Momentary Assessment (EMA) is increasingly used to collect real-time data on physical activity behavior. However, no activity-triggered EMA studies have been conducted among low-income older adults. We aim to assess the feasibility and acceptability of activity-triggered EMA in low-income older adults.

**Research Design and Methods:**

Researchers partnered with community organizations and provided technical support to facilitate the recruitment and retention of low-income older adults. For 7 days, 39 older adults (76.4 ± 8.5 years; 76% earning below £25 000/year) received EMA surveys when they surpassed a predefined activity/inactivity threshold, or when two hours elapsed between prompts. Participants wore a Move 4 activity sensor to measure their steps. A post-study questionnaire assessed perceptions of acceptability.

**Results:**

Participants completed 84% of the EMA surveys they received. EMA compliance did not appear to differ as a function of age, biological sex, time of day, day of week, or concurrent physical activity. Responses to the post-study questionnaire revealed that the most enjoyable aspects of participating in the study were making a worthwhile contribution to science and becoming more self-aware of their feelings and/or activities. The least enjoyable aspects included the frequency or timing of EMA prompts, the interruption of activities to complete an EMA survey, and limited or inadequate response options for certain EMA items.

**Discussion and Implications:**

Smartphone-based, activity-triggered EMA is feasible and acceptable among low-income older adults. Fostering community partnerships, managing participant resource constraints, and providing technical support are crucial strategies for engaging and retaining this population in mobile health research.

Innovation and Translational Significance: Ecological Momentary Assessment (EMA) can yield novel insights into health behaviors. This is the first study reporting the feasibility and acceptability of activity-triggered EMA among low-income older adults, who have the lowest levels of digital literacy. Feasibility was established by assessing EMA compliance, while acceptability was based on perceptions of study procedures. The integration of activity-triggered EMA with accelerometry was feasible and acceptable. This work could inform recruitment and retention strategies in mobile health research. Furthermore, the findings reinforce the potential of innovative EMA approaches for advancing our understanding of low-income older adults’ physical activity behavior.

## Background and objectives

Regular physical activity is associated with numerous benefits in older adulthood, including the prevention of cognitive decline, depression, frailty, and various comorbidities.^1^^,^^2^ Supporting older adults to partake in physical activity could not only improve their quality of life,^2^ but given the shift to an aging population, also help to drastically reduce health and social care expenditures.^3^ While population-based surveys show that individuals aged 60+ years are the least active segment of society,^4^ inequalities also exist across indicators of socioeconomic status (SES), with older adults of higher income (or lower deprivation) being more physically active than their lower income (or higher deprivation) counterparts.^5^^,^^6^ Understanding the factors that enable or hinder physical activity in diverse samples of older adults is therefore crucial for narrowing inequalities and improving overall health and well-being.

Studies exploring correlates of physical activity in lower-SES older adults have mostly relied on cross-sectional designs.^7^ However, qualitative research stipulates that many factors associated with physical activity are dynamic rather than stable over time and vary across contexts.^8^ Smartphone-based EMA overcomes some of the methodological weaknesses of existing literature, by gathering information on fluctuating behaviors, contexts, emotions, beliefs, and attitudes on a momentary basis in naturalistic settings.^9^^,^^10^ Activity-triggered EMA distributes surveys in response to participants meeting predefined activity or inactivity thresholds as determined through sensor technologies.^11^^,^^12^ Unlike interval- or signal-contingent sampling, which triggers EMA surveys at preset or random times throughout the day, this ensures that relatively infrequent behaviors, such as physical activity, are captured as and when they occur.^11^^,^^12^

Previous research has documented the suitability of smartphone-based EMA for understanding older adults’ physical activity behavior and the feasibility of deploying such protocols. Feasibility, in this context, has primarily been operationalized through compliance and retention rates, with some consideration given to the number and type of technical issues encountered by participants.^13–15^ Notably, high levels of data completion were reported in a 3-day activity-triggered EMA study among 69 well-educated older adults in Germany.^13^ Moreover, older adults participating in a 10-day study examining the feasibility and validity of signal-contingent EMA answered, on average, 92% (range 20–100%) of EMA prompts.^14^ Similar compliance with an 8-day random, interval-based EMA protocol was observed among low-income African American older adults.^15^ Importantly, participants considered the procedures acceptable and enjoyable, owing to the insights about oneself that it afforded.^16^ Despite these contributions, no empirical work has assessed the feasibility and acceptability of *activity-triggered* EMA among low-income older adults. In the United Kingdom, an age-related and socioeconomic divide persists with regard to technology adoption and use.^17^ Low-income older adults may therefore be excluded from mobile health studies, which frequently require access to a smartphone and/or a reliable internet connection.^18^ This needs to be addressed, as physical activity interventions and policies originating from evidence skewed toward more affluent individuals risk widening rather than narrowing health disparities.

Accordingly, this study aimed to determine the feasibility and acceptability of 7-day smartphone-based, activity-triggered EMA among low-income older adults. Feasibility was established by assessing EMA compliance and exploring whether this differed according to time-invariant and time-varying factors. Acceptability was gauged based on participants’ perceptions of the procedures with a focus on perceived burden and enjoyment.

## Research design and methods

### Participants

From October 2023 to March 2024, older adults aged 60+ years who self-reported being able to speak and read fluent English and living in South West England were recruited to participate in this study. Although we targeted older adults with an annual household income below £25 000 before taxes, those with a higher income recruited through networks with low-SES membership were also invited to participate. People were excluded if they were living in a care home, nursing home, or hospital; self-reported any functional limitations that prevented them from standing or walking on their own (individuals were eligible if they could only walk short distances or used an assistive device); were unable to see/utilize a smartphone’s basic functions; or had been diagnosed with dementia or Alzheimer’s disease by a medical practitioner. To access community-dwelling, low-income older adults, researchers partnered with gatekeepers at social housing associations (eg, almshouse charities) and community centers hosting events (eg, weekly lunch clubs or social groups) for older adults. The research team approached administrators, managers, wardens, team leaders, and/or coordinators of relevant organizations to determine the feasibility of conducting the study at their sites. Those interested in supporting the study served as a trusted conduit to residents or members, facilitating introductions with potential participants, sharing recruitment materials, and/or allowing the lead researcher to make recruitment announcements, as well as schedule study-related appointments to take place on-site (eg, in communal spaces), during upcoming meetings and events. Participants were also recruited through word-of-mouth, posters, patient and public involvement groups, and online adverts.

### Procedures

This study involved a single monitoring period lasting 7 days (5 weekdays and 2 weekend days). Eligible individuals were scheduled for an introductory session on Day 1. This was either held in a public place of the participants’ choice or in their own homes. At the introductory session, the lead researcher reviewed the procedures. Participants provided written informed consent and completed a baseline paper-and-pencil questionnaire assessing demographic information, physical activity habits, and smartphone usage. They were loaned a Move 4 activity sensor (movisens GmbH, Karlsruhe, Germany). If participants did not own a compatible mobile phone or preferred to use a separate mobile phone for the study, they were loaned a Motorola Moto G8 Power (Motorola, Inc., Chicago, IL) smartphone. Training included reviewing a “cheat sheet” provided to participants, which contained written and visual instructions for unlocking the smartphone, adjusting the volume, and answering/completing the EMA survey. If an EMA survey was delivered at the end of the introductory appointment or during an interim appointment, participants had the option of completing it as a practice survey with the research team. These responses were discarded from the analyses. Participants could contact the lead researcher via phone or email for technical support over the study period.

EMA surveys were delivered on the movisensXS application (version 1.5.23, movisens GmbH, Karlsruhe, Germany) for Android operating systems using event-based sampling and prompted by the following conditions (determined via the activity sensor): (1) surpassing a predefined activity threshold (>220 milli-g; 10-minute moving average), which is equivalent to a participant walking for at least 6 minutes during a 10-minute episode; (2) falling below a predefined inactivity threshold (<10 milli-g; 10-minute moving average), which is equivalent to minor movements during sitting episodes; and (3) two hours elapsing between prompts (ie, timeout prompts).^12^ The activity/inactivity thresholds were adaptive, such that momentary assessments were gathered during participants’ most and least active fragments of the day in a 1:1 ratio.^12^ There was no set frequency of prompts, so participants could receive different numbers of surveys. A minimum of 40 minutes was enforced between EMA prompts.

Participants received up to 18 items in each EMA prompt, assessing their current activity, context, feeling states, and motivation for engaging in physical activity over the next few hours. EMA surveys were delivered between 08:00 and 20:00, and each one was intended to take 1–3 minutes to complete. Participants were asked to carry their smartphones with them during all waking hours. An auditory signal and/or vibration alerted them when it was time to complete an EMA survey. If participants did not respond to the initial notification, they received four reminder notifications at 5-minute intervals, after which the EMA survey counted as missed. The activity sensor was clipped to a belt or the rim of trousers and placed on participants’ right hip (*N *= 26) or attached to the skin on their right thigh (*N *= 6; one participant wore it on their hip from Day 3), using waterproof adhesive film. For safety or comfort reasons, 7 participants wore it on their left hip. Participants were asked to wear the activity sensor during all waking hours if placed on the hip, or continuously (24/7) if placed on the thigh.

After 7 days, participants returned the equipment and completed a paper-and-pencil questionnaire assessing their perceptions of the study. They received a £20 shopping voucher for their participation, irrespective of EMA compliance, and were sent an optional individualized report from the activity sensor. All procedures were approved by the Biomedical Sciences Research Ethics Committee [0001-298] at the University of Bath. The Checklist for Reporting EMA Studies (CREMAS; see Supplementary Table 1) was followed.^19^

### Measures

#### Physical activity

Move 4 activity sensors, which have been validated in the general adult population,^20^^,^^21^ provided a device-based measure of physical activity. The activity sensor calculated movement acceleration (milli-g) in 30-second epochs. Each recording was date- and time-stamped to facilitate linkage with EMA data.^22^ Given that walking is the most common activity in older adults,^23^ concurrent physical activity was defined as the number of steps in the ±15-minute window around the EMA prompt. The DataAnalyzer software (version 1.15.1; movisens GmbH, Karlsruhe, Germany) calculated steps with a 60-second resolution.^12^ Observations where participants were not wearing the activity sensor for at least half of the 15-minute window before or after the EMA survey were excluded from the corresponding analyses.^15^

#### Demographic and temporal variables

Participants provided information including age and biological sex (male versus female) in the baseline questionnaire. EMA prompts were date- and time-stamped to allow the creation of temporal variables. Time of day was categorized as morning (reference group; EMA prompts between 08:00 and 11:59), afternoon (between 12:00 and 15:59), and evening (between 16:00 and 20:00). Day of week was dichotomized as weekday (reference group) or weekend day.

#### Study acceptability

Acceptability was assessed via the post-study questionnaire,^16^ containing four items asking participants to reflect on (a) the daily demands of the study, (b) the length of EMA surveys, (c) the experience of wearing the activity sensor every day, and (d) their willingness to participate in future research studies. Two open-ended questions asked about the most and least enjoyable aspects of participating in the study.

### Data analysis

Descriptive statistics and frequencies summarized EMA compliance (ie, the number of EMA prompts completed out of the total number delivered) and responses to the post-study questionnaire. Multilevel logistic regression models (observations nested within persons) explored time-invariant and time-varying factors influencing the odds of missing the EMA prompt. Completing the EMA prompt was coded as 0, and missing the EMA prompt (ie, not answering, or answering but not completing the EMA survey) was coded as 1. In Model 1, age, sex, time of day, and day of week were entered as independent variables. The number of steps in the ±15-minute window around the EMA prompt was added in Model 2 to test whether concurrent physical activity was associated with EMA non-response. Written answers to open-ended questions were manually tagged using a combination of deductive and inductive codes, which were then sorted into common categories by the lead researcher (O.S.M). Data were processed in Stata BE version 18.0 (StataCorp, College Station, TX), and multilevel models were performed in R version 4.4.1 with RStudio version 2024.04.2 using the *lme4* package.^24^

## Results

### Data availability and compliance

Of the 39 participants enrolled in the study (Table 1), 13% used their own mobile device and 87% were loaned a smartphone. EMA data were unavailable for two people due to premature uninstallation of the movisensXS application or turning off the smartphone and not receiving any surveys. One older adult dropped out on Day 3 due to participant burden but still completed the post-study questionnaire; their available data were retained in analyses. Across the remaining 37 participants, 1 998 EMA prompts were delivered, of which 1 672 were completed (84% compliance), 309 were not answered, and 17 were answered but incomplete. Participants completed, on average, 45 EMA surveys (standard deviation [SD] = 22) over the study period (range 1–72). On average, it took participants 2.0 minutes (SD = 3.7) to answer (ie, the difference between the time of the auditory signal and/or vibration and the time when the EMA survey was accessed by the participant), and 1.5 minutes (SD = 0.8) to complete (ie, the difference between the time when the EMA survey was accessed by the participant and the time when the EMA survey was completed), the EMA survey. The elapsed time, on average, between the auditory signal and/or vibration and completion of the EMA survey was 3.5 minutes (SD = 3.8). There were 290 observations without valid activity sensor data in the ±15-minute window around the EMA prompt. Based on the 1 708 observations with valid activity sensor data, participants took, on average, 578 (SD = 670), 65 (SD = 164), and 129 (SD = 261) steps in the ±15-minute window around activity-triggered (842 observations), inactivity-triggered (486 observations), and timeout (380 observations) prompts, respectively.

**Table 1.** Participant characteristics (*N *= 39).

Demographic variables	Mean (SD)	%
**Age (years)**	76.4 (8.5)	
**Body mass index (kg/m^2^)** ^a^	26.5 (4.6)	
**Biological sex (% male)**		41.0
**Ethnicity (% White)**		97.4
**Annual household income before taxes (% less than £25 000)** ^b^		75.7
**Current employment status (% retired)**		92.3
**Highest level of education completed (% university degree or higher)**		46.2
**Marital status (% married or living with a partner)**		20.5

*Note.* SD, standard deviation.

a Thirty-two participants had available self-reported height *and* weight data.

b Thirty-seven participants had available annual household income data (two participants responded “don’t know”), which included income from pensions.

### Predictors of EMA compliance

The results of multilevel models regressing EMA compliance on time-invariant (ie, age, biological sex) and time-varying (ie, time of day, day of week, concurrent physical activity) factors are presented in Table 2. Across both models, older age was marginally associated with higher odds of missing the EMA prompt. EMA compliance did not differ as a function of biological sex, time of day, or day of week (Model 1), or the number of steps in the ±15-minute window around the EMA prompt (Model 2).

**Table 2.** Associations of time-invariant and time-varying factors with EMA non-response.

Variable	**Model 1** ^a^	**Model 2** ^b^
	Odds ratio (95% CI)	Odds ratio (95% CI)
**Intercept**	0.00 (0.00, 0.46)^c^	0.00 (0.00, 0.22)^d^
**Age**	1.05 (0.99, 1.11)	1.06 (1.00, 1.12)
**Biological sex**		
** Male (reference)**	1.00	1.00
** Female**	1.05 (0.39, 2.84)	0.77 (0.29, 2.10)
**Time of day**		
** Morning (reference)**	1.00	1.00
** Afternoon**	0.84 (0.61, 1.17)	1.09 (0.74, 1.60)
** Evening**	0.93 (0.68, 1.28)	1.11 (0.75, 1.64)
**Day of week**		
** Weekday (reference)**	1.00	1.00
** Weekend day**	1.11 (0.83, 1.48)	1.11 (0.79, 1.55)
**Step count (±15-minute window)**	–	0.97 (0.83, 1.15)

*Note.* EMA, Ecological Momentary Assessment; CI, confidence interval. Concurrent physical activity (ie, the step count in the ±15-minute window around the EMA prompt) was standardized.

a Level 1: 1 998 observations; Level 2: 37 participants.

b Level 1: 1 708 observations; Level 2: 37 participants.

c *p *< .05.

d *p *≤ .01.

### Perceptions of study acceptability

The post-study questionnaire was completed by 38 participants (Table 3). Responses revealed that the daily demands of the study were reasonable (87% agreed or strongly agreed). Most participants rated the EMA surveys as being of reasonable length (87%) and disagreed or strongly disagreed (87%) that wearing the activity sensor was a nuisance. Furthermore, 89% of the sample responded “yes” to being contacted about future research studies.

**Table 3.** Responses to the post-study questionnaire (*N *= 38).

Item and response options	*N* (%)
**“The daily demands of the study were reasonable.” Please rate your level of agreement with this statement by ticking the appropriate box.**	
** Strongly disagree**	1 (2.63)
** Disagree**	0 (0.00)
** Neither agree nor disagree**	3 (7.89)
** Agree**	17 (44.74)
** Strongly agree**	16 (42.11)
** Prefer not to answer**	1 (2.63)
**“How did you feel about the length of the smartphone surveys?”**	
** Way too long**	1 (2.63)
** A little long**	1 (2.63)
** Reasonable length**	33 (86.84)
** A little short**	2 (5.26)
** Way too short**	0 (0.00)
** Prefer not to answer**	0 (0.00)
** Missing^a^**	1 (2.63)
**“Wearing the activity monitor everyday was a nuisance.” Please rate your level of agreement with this statement by ticking the appropriate box.**	
** Strongly disagree**	17 (44.74)
** Disagree**	16 (42.11)
** Neither agree nor disagree**	4 (10.53)
** Agree**	1 (2.63)
** Strongly agree**	0 (0.00)
** Prefer not to answer**	0 (0.00)
**“May we contact you about opportunities to participate in future research studies?”**	
** Yes**	34 (89.47)
** No**	1 (2.63)
** Prefer not to answer**	3 (7.89)

*Note.* The post-study questionnaire was not administered to one participant.

a This question was not applicable to one participant who didn’t receive/complete any EMA surveys.

Common responses to the open-ended question about the most enjoyable aspects of participating in the study were helping and contributing to something worthwhile (53%); becoming more self-aware of their feelings and/or activities (32%); and meeting the research team (13%). The least enjoyable aspects included the frequency or timing of EMA prompts (29%); the interruption of activities to complete an EMA survey (18%); limited or inadequate response options for certain EMA items (18%); difficulty using the smartphone (16%); and having to carry the smartphone and/or wear the activity sensor (16%). Moreover, 24% of participants did not respond to the question about the least enjoyable aspects of the study or mentioned that there was nothing they did not enjoy.

## Discussion and implications

This study explored the feasibility and acceptability of 7-day smartphone-based, activity-triggered EMA among predominantly low-income older adults. Overall, participants were compliant with the protocol, suggesting that low-income older adults are willing to participate in mobile health research and, when adequately trained, can complete an EMA study successfully.

Among the 37 participants with available data, 84% of EMA surveys were completed. This is consistent with EMA studies of physical activity or sedentary behavior in the general older adult population,^14^^,^^15^^,^^25–27^ which reported compliance rates of 73 to 93%. The level of data completeness is also similar to, albeit slightly lower than, an 8-day EMA study with six random prompts per day among low-income African American older adults, which reported 92% compliance.^15^ Discrepancies may be explained by differences in the intensity of the protocol, with 84% of participants in the current study receiving ten or more EMA prompts per day at least once during the study period. With respect to predictors of EMA compliance, evidence was inconclusive regarding whether non-response differed according to age or biological sex.^15^ Temporal variables were not associated with missing an EMA prompt, despite research showing that non-response tends to be higher in the afternoon compared to the morning, and on weekend days versus weekdays.^14^^,^^15^ These studies were conducted in the US, so may reflect cross-national variations in older adults’ health behaviors or routines.

Concurrent physical activity was unrelated to EMA missingness, suggesting participants were able to interrupt their activities to complete an EMA survey.^15^ In contrast, a sample of underweight and normal weight adults engaged in more moderate-to-vigorous physical activity in the 30 minutes surrounding unanswered versus answered EMA prompts.^28^ This is likely because activities of moderate-to-vigorous intensity are less conducive to carrying a smartphone and/or pausing to complete an EMA survey, relative to walking. Anecdotal evidence indicated that older adults occasionally missed EMA prompts during events such as going to the cinema, attending medical appointments, or visiting places of worship.

The procedures were deemed acceptable and enjoyable by low-income older adults. Despite the high frequency of EMA prompts, most participants rated the EMA surveys as being of reasonable length. These results align with low-income African American older adults’ perceptions of an 8-day EMA protocol.^16^ Participants also seemed satisfied with wearing the activity sensor. While non-wear could have been further curtailed by requiring participants to wear the activity sensor continuously,^15^ for added convenience, they could remove it overnight. Moreover, given that previous research has reported privacy or surveillance concerns as barriers to older adults’ use of digital health technologies,^29^ the research team clarified what data the activity sensor would (ie, physical activity) and would not (ie, location) record. Encouragingly, low-income older adults did not appear to experience more technical issues with the smartphone or activity sensor relative to other samples of adults or older adults.^14^^,^^16^^,^^28^

This work could inform strategies to boost engagement, compliance, and retention in mobile health research undertaken with low-income older adults. For instance, providing written and visual instructions, training participants at the introductory session, and establishing a helpline for technical support are likely to have contributed to the high compliance rate. While some researchers have relied on emails, websites, and social media to enroll older adults, our primary recruitment strategy involved partnering with wardens or team leaders in housing associations and third-sector organizations, who acted as gatekeepers. Furthermore, the researchers established trust and removed logistical barriers to low-income older adults’ participation, such as a lack of transport, by holding appointments in familiar public settings or their place of residence.

To our knowledge, this is the first study reporting the feasibility and acceptability of smartphone-based, activity-triggered EMA among low-income older adults. Establishing this in an underrepresented population is essential for developing effective and inclusive physical activity interventions, such as Ecological Momentary Interventions, which provide real-time, personalized behavioral support to users within their natural environments.^30^ However, there are limitations to acknowledge. First, although we overcame sampling bias toward higher socioeconomic groups by inviting older adults who did not own a smartphone to participate in the study, due to the small sample size, we were not able to evaluate differences in feasibility or acceptability measures by smartphone ownership. While some evidence suggests appraisals of study acceptability may be more negative among smartphone non-users,^16^ lending smartphones to participants could also increase engagement, due to the novelty of the devices. Further studies with larger samples could also investigate whether perceptions of wearing the activity sensor vary according to device placement.^31^ Importantly, there was no formal assessment of whether our attempts to reduce barriers to enrollment and support participant retention influenced the results. In addition, EMA surveys were delivered between 08:00 and 20:00, but some participants reported engaging in physical activity outside these hours. Although we may have missed some bouts of physical activity, our aim was to capture momentary “snapshots” of behavior rather than the total volume performed. Future research should employ qualitative interview methods to supplement responses to the post-study questionnaire and elicit more in-depth feedback from participants.

Our sample consisted primarily of low-income older adults; nonetheless, we acknowledge the variation present across other indicators of SES (eg, 46% of participants were university-educated). Despite their overlapping properties, income is a more contemporary and dynamic marker of SES in older age compared to education, which is often determined in early life and does not fully reflect evolving skill sets nor guarantee digital competence.^32^ Indeed, the digital revolution occurred well into the adulthood of many older individuals,^33^ and few participants in this study had prior experience using a smartphone. Moreover, income is associated with late-life health independently of other socioeconomic indicators, with a predictive value that is equal to, or even better than, that of a composite measure of SES (based on education, social class, occupational complexity, and income).^32^ Efforts were made to ensure that the sample in the current study was more socioeconomically diverse on the basis of income than what is typically found in EMA studies targeted at the general older adult population. However, further work adopting an intersectional approach is required to uncover how an older adult’s combination of identities across gender, ethnicity, disability, education, occupational class, income, and wealth influences their physical activity and digital participation, as well as to elucidate underlying mechanisms.

In conclusion, our results suggest that the integration of smartphone-based, activity-triggered EMA with accelerometry is feasible and acceptable among predominantly low-income older adults. This study reinforces the potential of EMA for advancing our understanding of low-income older adults’ physical activity behavior, while simultaneously highlighting the importance of diligent recruitment and retention efforts. Findings speak to low-income older adults’ willingness and ability to participate in mobile health research, particularly when their recruitment is facilitated by trusted gatekeepers, and they are provided with appropriate training and technical support from the research team. Determining the feasibility and acceptability of activity-triggered EMA in a frequently underrepresented population is a critical step toward the development of effective digital interventions promoting physical activity behavior.

## Supplementary Material

igaf151_Supplementary_Data

## Data Availability

The quantitative dataset underlying this article is available in the University of Bath Research Data Archive at https://doi.org/10.15125/BATH-01588. Due to privacy concerns, the data will be shared in a safeguarded and restricted way, by granting access only to bona fide researchers and upon request to the Research Data Archive. The qualitative data underlying this article cannot be shared publicly for the privacy of individuals who participated in the study. Processing and analytic code (Stata and R) are available in the GitHub repository at https://github.com/OliviaMalkowski/EMA-feasibility.git. This study was not preregistered.
